# Highly Precise Measurement of HIV DNA by Droplet Digital PCR

**DOI:** 10.1371/journal.pone.0055943

**Published:** 2013-04-03

**Authors:** Matthew C. Strain, Steven M. Lada, Tiffany Luong, Steffney E. Rought, Sara Gianella, Valeri H. Terry, Celsa A. Spina, Christopher H. Woelk, Douglas D. Richman

**Affiliations:** 1 University of California San Diego, La Jolla, California; 2 Veterans Affairs San Diego Healthcare System, San Diego, California, United States of America; George Mason University, United States of America

## Abstract

Deoxyribonucleic acid (DNA) of the human immunodeficiency virus (HIV) provides the most sensitive measurement of residual infection in patients on effective combination antiretroviral therapy (cART). Droplet digital PCR (ddPCR) has recently been shown to provide highly accurate quantification of DNA copy number, but its application to quantification of HIV DNA, or other equally rare targets, has not been reported. This paper demonstrates and analyzes the application of ddPCR to measure the frequency of total HIV DNA (pol copies per million cells), and episomal 2-LTR (long terminal repeat) circles in cells isolated from infected patients. Analysis of over 300 clinical samples, including over 150 clinical samples assayed in triplicate by ddPCR and by real-time PCR (qPCR), demonstrates a significant increase in precision, with an average 5-fold decrease in the coefficient of variation of pol copy numbers and a >20-fold accuracy improvement for 2-LTR circles. Additional benefits of the ddPCR assay over qPCR include absolute quantification without reliance on an external standard and relative insensitivity to mismatches in primer and probe sequences. These features make digital PCR an attractive alternative for measurement of HIV DNA in clinical specimens. The improved sensitivity and precision of measurement of these rare events should facilitate measurements to characterize the latent HIV reservoir and interventions to eradicate it.

## Introduction

Exponential amplification of nucleic acids by the polymerase chain reaction (PCR) is the cornerstone of modern molecular biology. Numerous methods have been developed to obtain quantitative information from PCR about the concentration of deoxyribonucleic acid (DNA) before amplification. The most widely used form of quantitative PCR (qPCR) is “real-time” PCR (qPCR), in which initial concentrations are extrapolated from sequential measurements during the cycling reaction [1]. Digital PCR (dPCR) [2], in which the amplification reaction is divided into thousands of microscopic reaction volumes, is a rapidly growing alternative that is potentially more accurate [3], [4], [5] and more precise [6], [7]. This paper focuses on the application of droplet digital PCR (ddPCR) [8], in which micro-partitioning is achieved by emulsification of the aqueous PCR reaction mixture in a thermostable oil.

Few fields have been more profoundly impacted by quantitative PCR than virology. The quantification and dynamics of HIV burden in infected patient were originally elucidated using quantitative PCR to measure viral ribonucleic acid (RNA) in blood plasma [9], [10],[9], [10] and viral load testing remains the standard clinical tool to assess the rate of disease progression. Combination antiretroviral therapy now results in suppression of plasma viremia below the level of detection of commercial assays in most treated patients, but HIV nucleic acids remain important indicators of residual infection in these patients. Proviral HIV DNA is the most widely used measure of cellular reservoir size, and other forms, including 2-LTR (long terminal repeat) circles and cell-associated RNA and DNA, provide additional information about viral dynamics.

Measurement of HIV DNA in translational research studies has relied upon a variety of home-brew assays [11], [12], [13], primarily based on real-time PCR [14], [15], [16]. Despite the importance of quantitative PCR assays in HIV research, the impact of assay variability is often ignored or underestimated. Both qPCR and most terminal dilution methods effectively measure logarithmic copy number. This approach improves dynamic range at the expense of accuracy and linearity [17]. Subtraction of measured values, often performed implicitly in longitudinal analyses or when comparing different DNA forms, further degrades the signal-to-noise ratio. This has resulted in data that are difficult to compare between studies, and at worst may be not robust or not informative due to dominant assay noise.

This paper describes an assay for HIV pol and 2-LTR circles in human peripheral blood samples based on droplet digital PCR. The assay relies on well-established biochemistry, using primer sets and TaqMan hydrolysis probes published previously [18], [19]. Quantitative benefits and limitations of this assay are analyzed and compared with a real-time PCR assay using identical primer/probe sets to illustrate the intrinsic advantages and limitations of these two assay formats.

## Materials and Methods

### Cell Samples

“Clinical samples” of HIV-1 seropositive patients analyzed had been previously collected in ongoing research studies approved by the applicable Institutional Review Boards at Brigham and Women's Hospital, Johns Hopkins University, the University of North Carolina, Chapel Hill, and the University of California, San Francisco. The authors were blinded to patient data for these samples. The cohorts from which these samples had been drawn were known. Most of these samples (>95%) were peripheral blood mononuclear cells, and the remainder consisted of purified CD4+ T cells. Purification of these CD4+ T cells had been performed at the collection site, and thus purification methods were not known.

“PBMC samples” were extracted from the blood of HIV-negative healthy donors at the University of California San Diego (UCSD). All subjects gave written, informed consent and blood collection protocols were approved by the UCSD Institutional Review Board. Peripheral blood mononuclear cells (PBMC) were purified from whole blood with Lymphoprep, following the manufacturer's recommended protocol.

“Infected CD4+ T cells” were purified from blood collected identically using a RosetteSep (StemCell Technologies) CD4+ T Negative Isolation Kit. Purified CD4 cells were incubated overnight prior to activation with surface-bound anti-CD3/anti-CD28 and infected with NL4-3 at a multiplicity of infection of 0.2. Two days after infection, cells were washed and cryo-preserved until DNA extraction.

### DNA Extraction and Digestion

Cellular DNA was extracted using a Qiagen DNA Blood Midi Kit, following the manufacturer's protocol. In all cases, DNA was ethanol precipitated following elution to increase concentration. The DNA concentration was estimated from the A_260_/A_280_ absorptivity ratio using a NanoDrop 2000 spectrophotometer (Thermo Scientific). When the DNA concentration was below the desired concentration for emulsification, the concentration was increased by ethanol precipitation and re-suspension. Where specified, templates were thoroughly mixed with background human genomic DNA obtained by identical extraction methods from HIV seronegative donors (“PBMC DNA”) or with sonicated salmon sperm DNA (Agilent Technologies).

Extracted DNA was heated to 95°C for 10 minutes then quenched on ice prior to digest with the restriction enzyme BSAJ-I (New England Biolabs) at 60°C for 1 hour.

### DNA Standards

Plasmids encoding the entire HIV genome (pNL4-3, AIDS Reference Research Reagent Repository) or a 2-LTR junction (a kind gift from Dr. Kristine Yoder, Ohio State University) were used as standards for qPCR.

### PCR Primers and Probes

Both real-time and digital PCR reactions used published primers to conserved regions of HIV pol (Hxb2 positions 2536–2662) [19] and to the HIV LTR (Hxb2 positions 9585–51) [18]. For real-time-PCR, genomic quantification used TaqMan Ribonuclease P (RNase P) Control Reagents (Life Technologies). For ddPCR, an RPP30 (RNAse P) primer/probe set was used for host genomic DNA quantification. Samples were diluted 10-fold and RPP30 was assayed without multiplexing. Primers and probes are listed in Table S1.

### Real-Time PCR

Real-time PCR reactions were performed in a 50 µL solution containing 25 µL PerfeCTa Multiplex Master Mix containing ROX (Quanta Biosciences), 900 nM primers, 250 nM probe, and 1 µg of template DNA. Reactions were conducted in triplicate. Cycling proceeded in an Applied Biosystems 7900HT Sequence Detection System with the following parameters: 50°C for 2 minutes, 95°C for 10 minutes, followed by 50 cycles of 95°C for 15 seconds and 60°C for 1 minute. A standard curve of pNL4-3 plasmid DNA in triplicate, 10-fold serial dilutions was included on each plate. PCR reactions used FAM-labeled pol probes multiplexed with VIC-labeled RNAse P for genomic DNA quantification, together with the corresponding forward and reverse primers. A FAM-labeled 2-LTR probe and 2-LTR primers were used in a separate reaction for each sample.

### Digital PCR

The PCR reaction mixture was loaded into the Bio-Rad QX-100 emulsification device and droplets were formed following the manufacturer's instructions. The contents were transferred to a 96-well reaction plate and sealed with a pre-heated Eppendorf 96-well heat sealer for 2 seconds, as recommended by Bio-Rad.

Total DNA was amplified separately in an Applied Biosystems GeneAmp 9700 thermal cycler. Each reaction consisted of a 20 µL solution containing 10 µL ddPCR Probe Supermix, 900 nM primers, 250 nM probe, and template DNA with the following cycling conditions: 10 minutes at 95°C, 40 cycles each consisting of a 30 second denaturation at 94°C followed by a 58°C extension for 60 seconds, and a final 10 minutes at 98°C. After cycling droplets were analyzed immediately or stored at 4°C overnight and until analysis.

### Data Analysis

Raw fluorescence data for each well were exported from the manufacturer's software (Bio-Rad QuantaSoft v. 1.2) for analysis. Analysis of individual event data was performed using custom software written in Mathematica 8.0 (Wolfram Research). Droplets were classified as positive, negative or ambiguous following a custom algorithm that filters out potentially spurious events. (Fig. S5) Events that remained ambiguous after all data processing were excluded.

The number of template copies per unit volume *μ* was estimated from the number of positive events *n* detected in the corresponding channel and the number of total accepted droplets *N* by maximum likelihood. The distribution of templates within a drop was assumed to follow a Poisson distribution, and the number of positive droplets was assumed to follow a binomial distribution. Confidence intervals were estimated under the same assumptions. The droplet size was assumed to be 0.91 nl, consistent with the instrument manufacturer's software.

Template copies per sample were computed by averaging over all available replicate wells. Total cellular DNA input was measured by halving the estimated number of RPP30 copies, and copy numbers per diploid cell equivalent were computed as the ratio of template (pol or 2-LTR) copies per diploid cell.

Statistical analyses were performed using Graph-Pad Prism 5.0 software (GraphPad Software, Inc.) and Mathematica 8.0.

## Results

**Figure 1:**
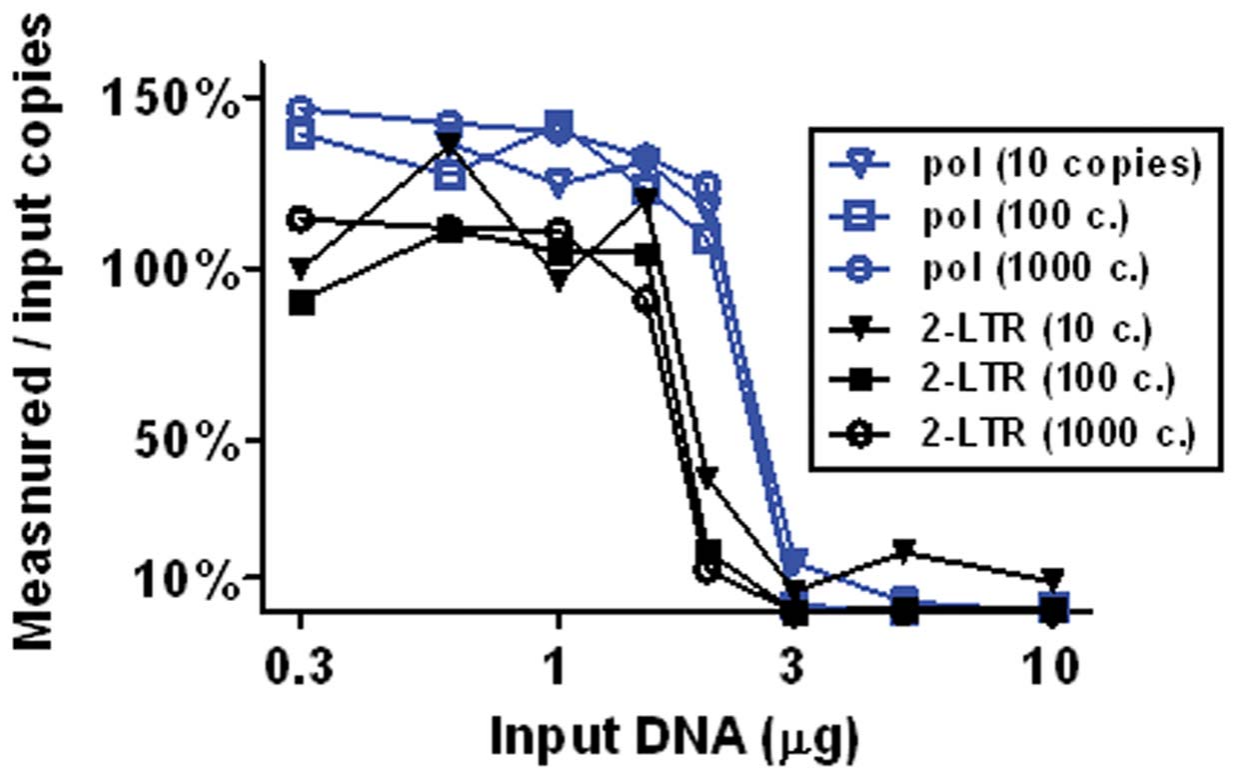
PCR Inhibition by background host DNA. Plasmids containing pol (pNL4-3) or the 2-LTR junction sequence were spiked into varying amounts of cellular background (salmon sperm) DNA prior to droplet formation. The figure shows the measured copy number as a percentage of the value expected from A_260_/A_280_ absorptivity of the plasmid. Loading of over 1.5 µg of DNA per well greatly reduced the measured copy number for all samples tested. Above 3 µg/well, droplet formation was also inhibited (not shown).

It was desirable to load as much DNA as possible into each well to maximize assay sensitivity, because the HIV targets of interest are extremely rare amidst a large background of host cellular DNA. To determine the maximum amount of cellular DNA that could be loaded into a single well, 10, 100 or 1000 copies of pNL4-3 or 2-LTR plasmid were spiked into a background of salmon sperm DNA prior to droplet formation (Fig. 1). Measured copy numbers did not vary significantly when up to 1 μg of background cellular DNA was loaded. A sharp decrease in measured copy numbers was noted when more than 1.5 μg was loaded per well, consistent with inhibition of PCR amplification. When more than 3 μg was loaded into a single well, the number of droplets formed decreased. In some cases, gross deformation of formed droplets was visible to the naked eye. Based on these results, subsequent experiments loaded 1 μg of DNA in each well, except as noted below.

**Figure 2:**
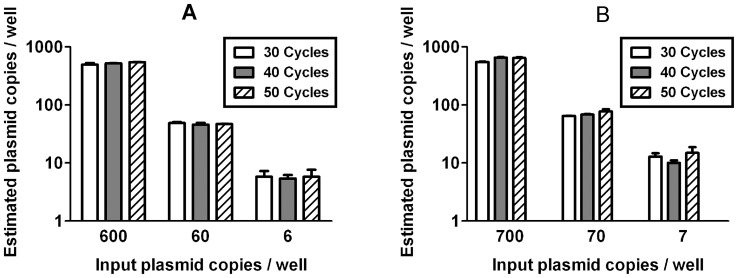
Values measured by digital PCR do not vary with cycle number. Plasmid templates were emulsified into droplets and thermally cycled for 30 to 50 cycles before analysis. No significant differences in measured *pol* (**A**) or 2-LTR (**B**) copy numbers were observed over this range of cycling times. No positive events were observed after 20 cycles (not shown). Similar results were obtained using dilutions of infected CD4+ T cells into uninfected PBMC (Fig. S.1). Error bars indicate the observed standard deviation between wells.

The Bio-Rad digital PCR system measures only endpoint droplet fluorescence after all thermal cycling is completed. Thus measured copy numbers might vary with the number of amplification cycles used. For perfectly efficient amplification, a single starting template would be amplified to the probe concentration (250 nM) in 25 cycles, resulting in signal saturation. To test the sensitivity of ddPCR to variation in cycle number and to determine the optimal cycle number, test samples consisting of plasmid (Fig. 2) or diluted, infected CD4 cells (Fig. S1) were divided between three plates and cycled for 30, 40 or 50 cycles. No significant changes were noted within this range. When the cycle number was reduced to 20, the signal was completely eliminated and no positive events were detected (data not shown). Subsequent experiments were conducted using 40 PCR cycles.

**Figure 3:**
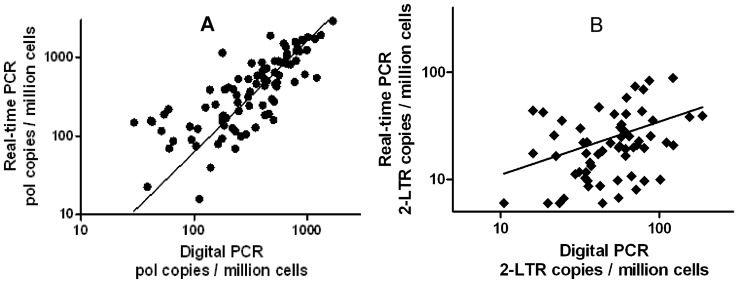
Correlation of ddPCR and qPCR measurements. (**a**) Pol copy numbers measured by ddPCR and qPCR were significantly correlated (Pearson R^2^ = 0.64, slope = 0.98±0.08). The correlation weakened at low copy numbers, primarily due to a rapid increase in the variance of the qPCR assay (Fig. S3 (a)). For copy numbers measured in the bottom tertile (<230 HIV DNA copies/10^6^ cells) by ddPCR, the correlation was not significant (R^2^ = 0.08, P = 0.12). In the central tertile, the correlation was weak but significant (R^2^ = 0.15, P = 0.03). In the top tertile, the correlation was strong (R^2^ = 0.53, P<0.0001). (**b**) 2-LTR copy numbers measured by both methods were significantly but weakly (R^2^ = 0.14, P = 0.002) correlated.

To compare the accuracy of the HIV DNA quantification by the two methods, stored PBMC samples (*N* = 156) were analyzed by both qPCR and ddPCR in triplicate. Samples were collected from 24 patients at varying times during the first 6 months following initiation of combination antiretroviral therapy (cART). Measurements made by the two methods were linearly correlated (Pearson *R* ^2^ = 0.64), with a slope statistically indistinguishable from unity (est. slope  = 0.98±0.08, Fig. 3). The correlation weakened at lower copy numbers, and was not significant in the bottom tertile (<230 copies per million PBMC).

**Figure 4:**
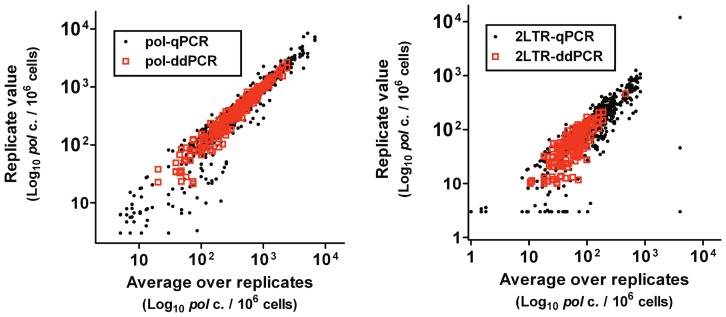
Precision of ddPCR versus qPCR. Samples of DNA (*N* = 156) isolated from the PBMC of patients during the first 6 months of cART were analyzed by both qPCR and ddPCR in triplicate. The reproducibility was higher for both the pol (**a**) and 2-LTR (**b**) targets. Because the noise and stochasticity of all assays varies with copy number, the relative precision was assessed by averaging the coefficient of variation within small bins (Fig. S2 (b)). The average coefficient of variation was 4-fold lower by ddPCR (Fig. S4 (b)) and 20-fold lower for 2-LTR (Fig. S4 (c)). An imputed value of 6 copies/10^6^ cells, equivalent to 1 copy/well, was used for wells in which no template was detected.

Although levels of cell-associated HIV DNA are affected much less by cART than is plasma HIV RNA, levels of residual HIV DNA after several years of suppressive therapy are significantly lower than during the first 6 months of therapy [20]. To evaluate the precision of the digital PCR assay for samples collected from patients on suppressive cART, PBMC samples (N = 146) from 3 distinct cohorts of patients were analyzed by ddPCR in triplicate (Fig. 4). All patients were enrolled in studies requiring undetectable viremia for at least 6 months. To assess the intrinsic accuracy of the assay, pol and 2-LTR concentrations estimated within each well were compared with the result averaged over triplicate wells (Fig. S2 (a)). As expected, assay variability increased at lower template concentration.

The specific mathematical form of this expected variation among replicates is a binomial distribution
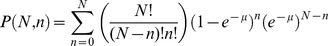
Where 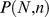 is the probability that *n* of *N* droplets is positive and µ is the unknown template concentration before droplet formation. In the limit of large *N*, this is approximated well by a Poisson distribution. To test the hypothesis that detected templates are Poisson distributed, the data were grouped into bins of width 0.1 log_10_ bins. The average coefficient of variation (C.V.) was computed for each bin, and these averages were fitted by linear regression (Fig. S2 (b)). The C.V. increased as the template number to the 0.48±0.07, consistent with a Poisson distribution.

To determine the distribution of assay noise in the qPCR assay, plasmid or infected cell templates were serially diluted and evaluated by qPCR with 10–12 replicates per sample (Fig. S3). For template frequencies above 300 copies/million cells, corresponding to a cycle threshold (C_T_) of 32 or fewer, the coefficient of variation was low and independent of template concentration. In this regime, the error distribution was approximately log-normal with a standard deviation of 0.07 log_10_ copies.

At low template concentrations, random sampling of templates occurs in qPCR, suggesting that the noise distribution should become Poissonian in this regime. However, the ratio between the copy number estimated per well and number of events N in the Poisson distribution might not be the expected value, primarily because the absolute copy number estimates rely on a (potentially skewed) standard curve. The coefficient of variation was therefore fitted to a model that assumed log-normal errors at high copy number and Poisson distribution at low copy number (Fig. S3 (a)). The increase in assay noise is consistent with Poissonian variation, but the constant ratio exceeded the expected value over 10-fold (maximum likelihood estimate  = 23×). Thus the qPCR assay errors are comparable to the ddPCR errors at 23-fold higher template concentration.

**Figure 5:**
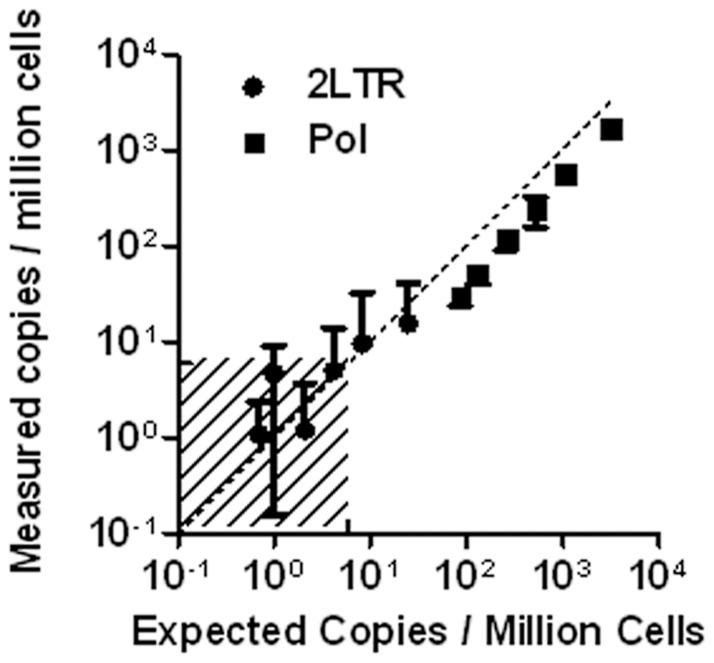
Assay limit of detection and quantification. CD4 cells infected in vitro were serially diluted into a background of PBMC DNA. The number of replicate wells was increased from 3 to 36 in proportion to the dilution, allowing accurate measurement below one copy per 10^6^ cells. The shaded area indicates concentrations below the theoretical limit of detection for a single well.

Next, the limit of detection of the ddPCR assay was evaluated. The assay used in previous experiments loaded DNA from approximately 500,000 cells into each triplicate, making detection of templates present at fewer than 2 copies per million theoretically impossible. To differentiate the intrinsic detection limit for the ddPCR assay from this loading limit, a serial dilution of DNA isolated from infected CD4 cells into uninfected PBMC DNA was tested with a number of replicate wells that increased as the reciprocal of the template number to a maximum of 36 wells (Fig. 5). This allowed detection of both pol and 2-LTR circles at frequencies of 0.7 copies per 10^6^ cells.

All experiments described above used the same conserved primer-probe set. Because of the high diversity of HIV genomes in vivo, even regions that are relatively well conserved may vary in some patients. Mismatches in the primers or the probes might reduce the efficiency of amplification or probe hydrolysis without eliminating the signal completely. In this scenario, qPCR may substantially underestimate the true template copy number. This problem can be circumvented by using patient-specific primers and probes if HIV DNA sequence data are available, but this approach is cumbersome [19].

**Figure 6:**
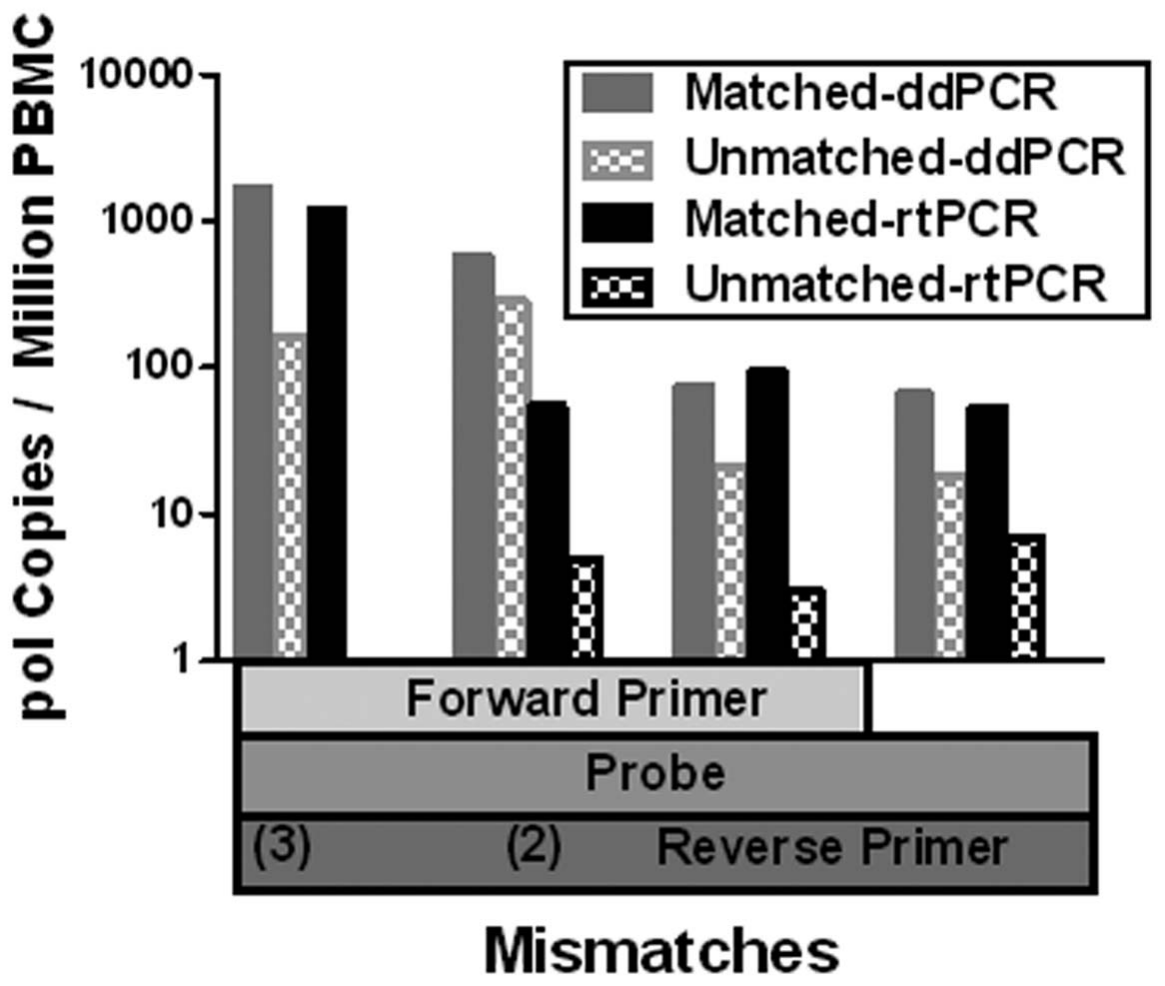
Effect of sequence variation. Patient isolates with previously determined *pol* sequences that differ from the consensus primer/probe set in at least two positions were analyzed by ddPCR and by qPCR. Both assays were conducted in parallel using the mismatched consensus primer/probe set and a patient-specific matched primer/probe set. Use of consensus primers and probe resulted in an underestimate of copy number by one to two log_10_ by qPCR, with complete loss of detection in the extreme case of 5 total mismatched bases. The underestimate was largely mitigated (mean 57% reduction in log_10_ copy number change) by ddPCR in all cases. These 4 cases reflect the most extreme mismatches observed in 84 patients, suggesting that sequence variation is unlikely to significantly impact ddPCR assay results in clinical studies. All samples analyzed were HIV-1 subtype B.

To determine how ddPCR measurements would be affected by sequence variation, samples were selected from a large cohort with pol sequence data available. Among these 84 patients, 21 had variations in the sequence complementary to the probe and over half had variations at one or both primer sites. Four patients with the most extreme mismatches between pol sequence and the consensus primer/probe set were evaluated by both ddPCR and qPCR using both patient-specific and consensus primers and probes (Fig. 6). With qPCR, the use of consensus sets resulted in an underestimate of pol concentration by 10 to 100-fold in 3 of the 4 patients. In the fourth patient, no template was detected at all using consensus primers. When the same samples were analyzed by ddPCR, the underestimate (in log_10_ pol c/10^6^ cells) was reduced in all cases, with an average 57% reduction.

## Discussion

Several applications of digital PCR reported recently have demonstrated that the high accuracy possible in theory can be practically realized with a variety of clinical specimens [4], [7], [21], [22], [23], [24]. The need for a standardized, accurate assay for persistent HIV DNA in infected patients led us to develop a ddPCR assay for HIV DNA. This need is especially relevant to investigate the latent HIV reservoir. The extremely rare HIV DNA targets studied here pose unique challenges, and the theoretical performance of both qPCR and ddPCR assays might diverge from results obtained in analysis of clinical specimens for such rare targets. We therefore optimized and characterized the performance of both assays in detail.

To maximize assay sensitivity, the maximum input DNA concentration was established first. Inhibition of the PCR reaction occurred abruptly when over 1.5 μg of extracted DNA was loaded into the 20 μl reaction input. (Fig. 1) Although this inhibition was expected, the exact mechanism is unclear. Droplet formation was noticeably impaired at loading concentrations as low as 3 μg per 20 μl, suggesting that chemical reactions at the oil-water interface of the emulsion may play a role.

The number of amplification cycles used had no discernible effect on measured values of either pol or 2-LTR, provided that at least 30 cycles were used. (Fig. 2 and Fig. S1) Twenty PCR cycles, which would yield only 1.5 nM amplicon from a single template with perfect amplification efficiency, produced no discernible change in fluorescence above background. The robustness of event counts with varying cycle number justifies the use of endpoint fluorescence, which is necessary with the digital droplet platform used in this study (Bio-Rad QX-100). Real-time monitoring of fluorescence during thermal cycling is possible with other hardware configurations, but we could not evaluate whether this would this would provide any benefit due to system hardware limitations.

Because identical primers and probes were used for the two assays, we expected both assays to provide similar information. To determine the correlation and bias of the two methods, 156 clinical samples were tested by both assays. Values measured by the two techniques were strongly correlated (*R* ^2^ = 0.64), and there was no discernible bias between the two methods. Therefore both assays indeed provide similar measurements, and the principal difference between them is the structure and magnitude of noise.

The primary motivation for developing a ddPCR assay for HIV DNA was the prospect of reducing the noise observed in the qPCR assay. Three different methods were used to determine assay accuracy. As an initial test, plasmids or patient samples were serially diluted and evaluated using a large number of replicates. (Figs. 4 and S3(b)) Next, the variance among replicates was compared using the 156 samples assayed in parallel by both methods. (Fig. 3) Finally, an independent set of 214 clinical samples drawn from 3 different cohorts was analyzed by ddPCR, and the variance among triplicate wells for each sample was evaluated. (Fig. S2 (a))

All three methods led to the conclusion that ddPCR was substantially more precise for HIV template frequencies below 300 copies/10^6^ cells. For ddPCR, the variance among replicates approached the mean over replicates. This is consistent with the hypothesis that Poissonian variation due to random sampling is the principal source of noise in ddPCR assays of rare templates [4], [8], [25]. The variance also increased linearly with the mean for the qPCR assay, but the magnitude of this variance was higher by an average of 4-fold for pol (Fig. S4 (b)) and by 20-fold for 2-LTR (Fig. S4 (c)). This suggests that a Poissonian process produces accuracy-limiting noise in the qPCR assay, as well, but the process is evidently not simply random sampling of template by pipetting into the 96-well reaction plate.

It is unclear why the real-time 2-LTR assay was so much less precise. It had previously been noted that pre-heating samples improved the performance of the 2-LTR assay (data not shown). This procedure, which has been speculated to remove secondary structure, was used consistently here. Samples were not digested with a restriction enzyme prior to real-time analyses, so it is possible that the restriction digest used prior to digital PCR had the inadvertent benefit of removing inhibitory secondary structure in episomal DNA.

The term “limit of detection” is used in various ways depending on the context. The definition that seems most applicable in typical HIV research applications is simply the template concentration below which detection will usually not occur at levels distinguishable from background noise. In the triplicate format used predominantly here, this limit is between 3 and 4 copies per million cells, depending on the number of droplets formed. A far more mathematically demanding definition used in clinical chemistry requires that the 95% confidence intervals of an undetected sample not overlap with the 95% confidence interval of a sample at the limit of detection [26]. By this definition, the limit of detection would be 14 copies per million cells. This latter definition might indeed be more appropriate for clinical diagnostic use in which disregarding a “zero” value could lead to inappropriate clinical management, but that is not the intended use here. The behavior of the assay near the limit of detection is consistent with Poisson statistics, and the limit of detection can therefore be calculated by any definition desired.

As shown in Fig. 5, the limit of detection can be further decreased by increasing the number of replicates analyzed. Note, however, that false positive background events preclude arbitrary improvement in the limit of detection. False positive events in no-template control wells, while infrequent as a percentage of all droplets analyzed, were observed regularly. In general, these were not distinguishable from true positives based on fluorescence data. (Figs. S6 and S7) Retrospective analysis of over 500 no-template control wells estimated an average of between 0.1 and 0.4 false positive events per well, and somewhat higher rates have been reported previously [8]. This average rate appeared to fluctuate over periods of several weeks (data not shown), but the source of false positives is unclear. Based on this background rate, our absolute limit of detection has varied from 0.7 to 3 copies per million cells.

One important limitation of the Bio-Rad QX-100 system is the dynamic range. Because the system works by counting positive reactions among approximately 15,000 droplets per sample well, the dynamic range is limited to between 4 and 5 log_10_ [8]. Thus HIV DNA copy numbers per cell cannot be measured in a single well by ddPCR. Either the HIV target is too scarce to detect or the host gene used for normalization (here RPP30) will is too abundant to quantify without saturation. This limitation can by circumvented by combining dilution series with ddPCR. The assay described here required used a single 10-fold dilution (in triplicate) to address this problem. Other implementations of digital PCR have a different dynamic range. It is therefore difficult to generalize about the linear dynamic range of our digital PCR assay, since this could easily be extended with additional dilutions.

The ddPCR work described here used the Bio-Rad QX-100 droplet digital PCR system exclusively, and our results may not apply to alternative implementations of digital PCR. The current QX-100 system suffers from two limitations that significantly impact the HIV DNA assay reported here. The first is the unexplained false positive events mentioned above. In addition, categorization of events as positive or negative based on measured fluorescence values is not trivial. The default thresholds set by Bio-Rad's QuantaSoft analysis software result in some obviously incorrect calls. Re-analysis of raw fluorescence data using custom software corrected most of these errors, but false positive could not be completely eliminated. (Figs. S6 and S7) Whether these important system limitations are intrinsic features of the QX-100 platform or can be eliminated with future improvements to the system software, device hardware, disposable components or assay protocols is currently unclear.

Despite these limitations, droplet digital PCR was found to be a practical method for highly accurate measurement of HIV DNA targets in clinical specimens. Existing qPCR assays for pol and 2-LTR HIV DNA were readily adapted to digital PCR without modification of primer/probe sets, re-optimization of thermal cycling parameters or changes in clinical sample processing. Unexpectedly, ddPCR also proved more robust to target sequence variation. Most importantly, the improved accuracy and precision resulted in reliable quantification of proviral and episomal HIV DNA targets well below the limit of quantification of qPCR. These features make ddPCR particularly well suited to measurement of the size of the HIV latent reservoir and suggest that this assay could prove useful for clinical studies aimed at eradication of HIV from infected patients.

## Supporting Information

Figure S1 **Values measured by digital PCR do not vary with cycle number.** DNA isolated from CD4+ T cells infected in vitro was serially diluted, emulsified into droplets and thermally cycled for 30 to 50 cycles before analysis. No significant differences in measured pol or 2-LTR copy numbers were observed over this range of cycling times. No positive events were observed after 20 cycles (not shown). False-positive events in no-template control wells were also unaffected by cycle number (data not shown).
(PDF)

Figure S2 **Poissonian noise among ddPCR replicates.** (**a**) Copy numbers estimated from each well are plotted versus the average over triplicate wells for 370 clinical samples. In addition to the 156 samples described All patients were on cART and were enrolled in studies requiring at least 6 months of suppressed plasma viremia (<50 HIV RNA copies/ml). Wells with no events detected are excluded from the plot in (**a**), but not from the analysis in (**b**). Red dashed lines show the expected value plus or minus one standard deviation, assuming the data are Poisson distributed. (**b**) To more rigorously assess whether the data in (**a**) are consistent with a Poisson distribution, the coefficient of variation was computed for each well (black dots), and errors were averaged over bins of width 0.2 log_10_ (blue squares). Observed average errors (solid black regression line) were smaller than the Poissonian prediction (dashed violet line) by 0.15±0.10 log_10_, but this difference was not statistically significant.
(PDF)

Figure S3
**Noise in qPCR assay.** The cycle threshold (C_T_), which measures the number of PCR cycles required for template amplification, varies only slightly (median 0.07 cycles) at low C_T_ values (higher target amounts). Templates consisting of either plasmid or samples from an HIV-infected patient were diluted into HIV- cellular DNA background to determine the effect of input copy number on assay variability. (**a**) As the number of input template copies decreases below about 300 copies/10^6^ cells, the standard deviation of the C_T_ value among replicate wells increases rapidly. Poissonian sampling noise (dotted line) imposes a theoretical minimum on the assay noise (dotted line), but the observed noise (dashed line) is much larger (least-squares fit  = 23× larger). (**b**) The corresponding number of pol copies *N* estimated based on the best fit to a standard curve (
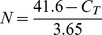
) illustrates the large variance compared to ddPCR (Fig. S2(a)), even for plasmid templates.(TIF)

Figure S4 **The ddPCR assay is more precise, particularly for 2-LTR circles.** Samples isolated from the PBMC of infected patients were assayed by both methods. To compare the precision throughout the tested range, the C.V. was averaged over bins as in Fig. S2 (b). All 370 clinical samples analyzed by ddPCR in Figs. 3 and S2 (a) were included, despite the fact that qPCR data was only available for the 156 samples shown in Fig. 3. (**a**) For both methods and both targets, the C.V. increases with a slope statistically that is not significantly different from ½, the expected value for Poisson-distributed noise (dashed line). Trend lines computed independently for the four assays did not have distinguishable slopes. Therefore, in order to estimate the average relative precision, maximum likelihood fits shown assume the ½ exponent. The offset between the trend lines indicates the relative precision. (**b**) For the pol target, ddPCR is 4-fold more precise. (**c**) The precision improvement is much greater for the 2-LTR target, with an average 20-fold improvement over qPCR.
(PDF)

Figure S5 **Triage classification of events.** Raw fluorescence data were first filtered to eliminate events consistent with irregular droplet size (“rain” and “hail”). The remaining events were analyzed according to the algorithm shown. First, the largest droplet clusters were identified. In most cases, positive events were rare and nearly all events were associated with droplets that were negative in both fluorescence channels. Significant clusters were then approximated with a binormal distribution, and the probability of each droplet was determined for each of these distributions. Events that were highly unlikely within any of the binormal distributions were classified as ambiguous. Finally, independent assortment of the duplexed targets was used to eliminate events with an unlikely combination of fluorescence amplitudes. Restriction enzymes used in this study were always expected to cut between the duplexed amplicons, so the number of positive events in each channel was assumed independent. This was used to identify spurious double-positive events.
(PDF)

Figure S6 **Sample dot-plots illustrate threshold ambiguities in ddPCR.** Raw fluorescence values from a single well are shown. Default thresholds set by Bio-Rad QuantaSoft analysis software (version 1.1) are shown as colored rectangles, and the corresponding event counts in each quadrant are shown. (**a**) “Rain” and “hail” extend outward from the central peak of dual-negative events, but these are easily distinguished from true positive events by cluster analysis. (**b**) The pattern is similar to (a), but the sparseness of hail complicates its discrimination from true positive events. The one dual-positive event called by QuantaSoft can be eliminated by the assumption of independent assortment.
(PDF)

Figure S7 **Sample false positives in ddPCR.** Positive events called in no-template control wells may reflect erroneous calls by the default thresholding algorithm (**a**), but most false-positive events are well separated from true negative events and thus cannot be identified mathematically (**b**).
(PDF)

Table S1 **PCR Primers and Probes.**
(DOCX)

Table S2 **Summary of Clinical Characteristics for Unblinded Patients.** The 156 samples analyzed by both qPCR and ddPCR were drawn from ACTG5248, during the first 6 months of treatment.
(DOCX)
