# Supplementary figures and images for: Highly Precise Measurement of HIV DNA by Droplet Digital PCR
Source: PLoS One. 2013 Apr 3;8(4):e55943. doi: 10.1371/journal.pone.0055943 (PMC3616050; doi:10.1371/journal.pone.0055943)

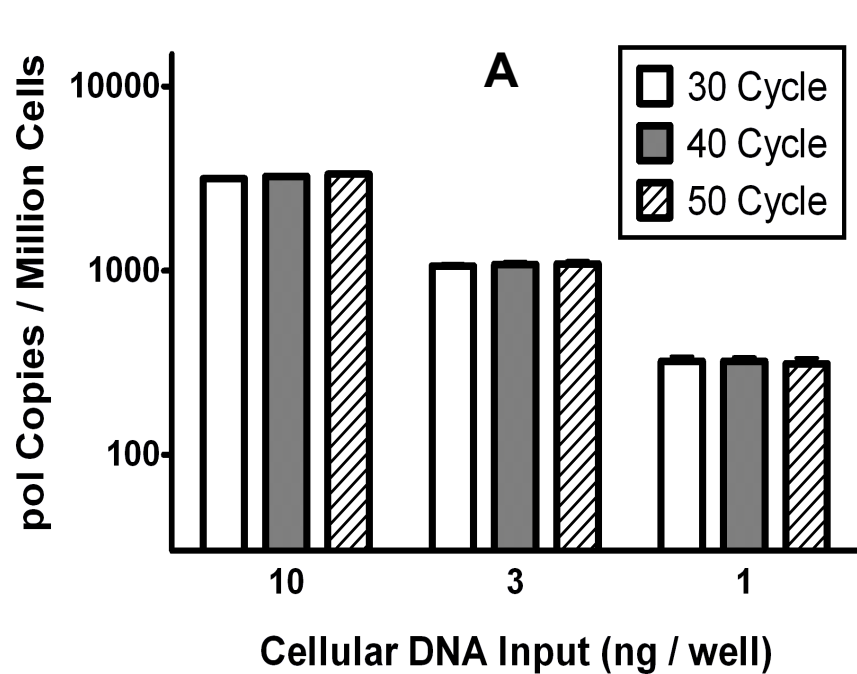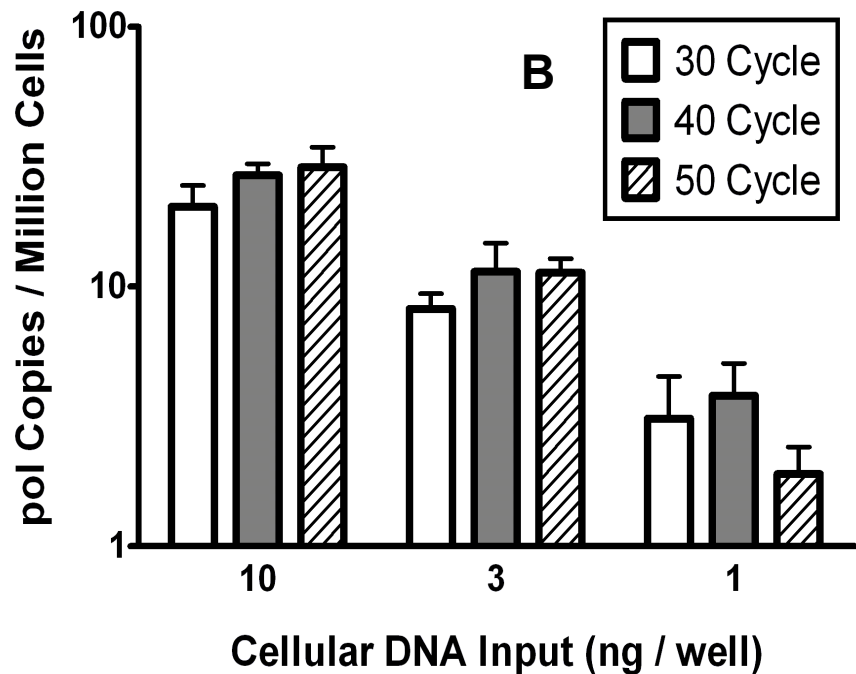

Supplement: Figure S1 — Values measured by digital PCR do not vary with cycle number. DNA isolated from CD4+ T cells infected in vitro was serially diluted, emulsified into droplets and thermally cycled for 30 to 50 cycles before analysis. No significant differences in measured pol or 2-LTR copy numbers were observed over this range of cycling times. No positive events were observed after 20 cycles (not shown). False-positive events in no-template control wells were also unaffected by cycle number (data not shown). (PDF) [file pone.0055943.s001.pdf]

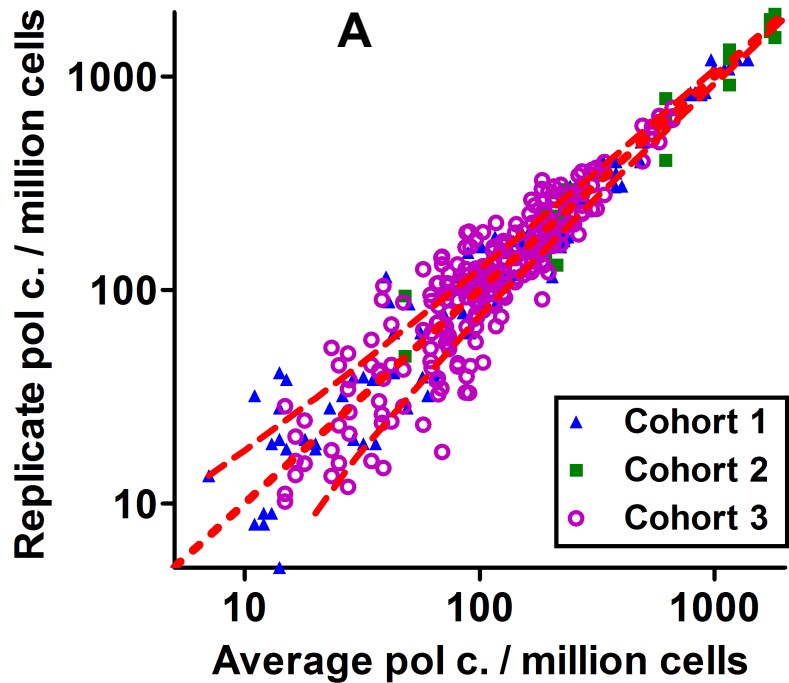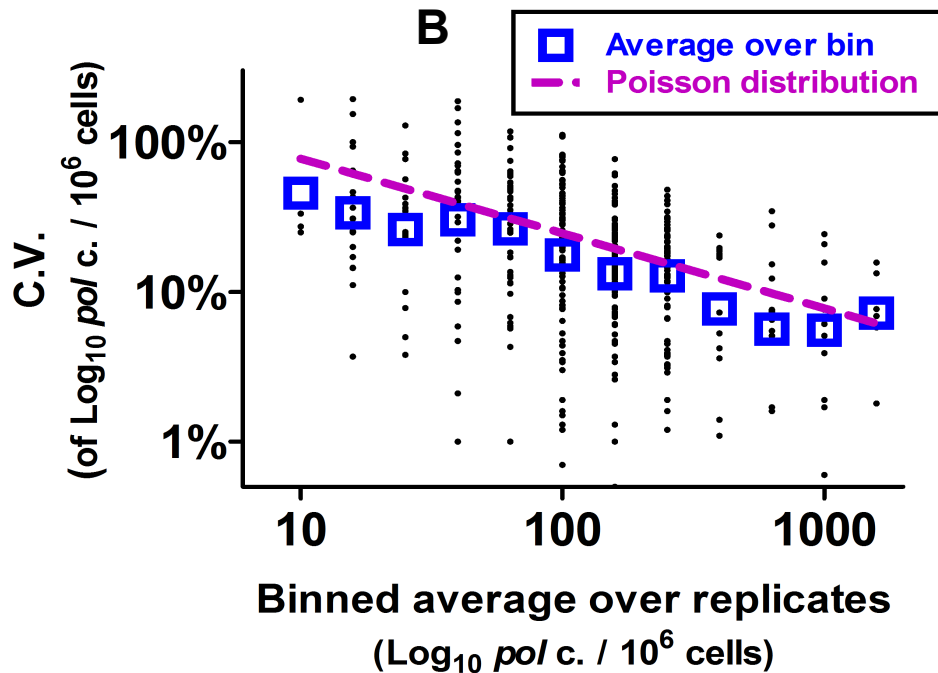

Supplement: Figure S2 — Poissonian noise among ddPCR replicates. (a) Copy numbers estimated from each well are plotted versus the average over triplicate wells for 370 clinical samples. In addition to the 156 samples described All patients were on cART and were enrolled in studies requiring at least 6 months of suppressed plasma viremia (<50 HIV RNA copies/ml). Wells with no events detected are excluded from the plot in (a), but not from the analysis in (b). Red dashed lines show the expected value plus or minus one standard deviation, assuming the data are Poisson distributed. (b) To more rigorously assess whether the data in (a) are consistent with a Poisson distribution, the coefficient of variation was computed for each well (black dots), and errors were averaged over bins of width 0.2 log10 (blue squares). Observed average errors (solid black regression line) were smaller than the Poissonian prediction (dashed violet line) by 0.15±0.10 log10, but this difference was not statistically significant. (PDF) [file pone.0055943.s002.pdf]

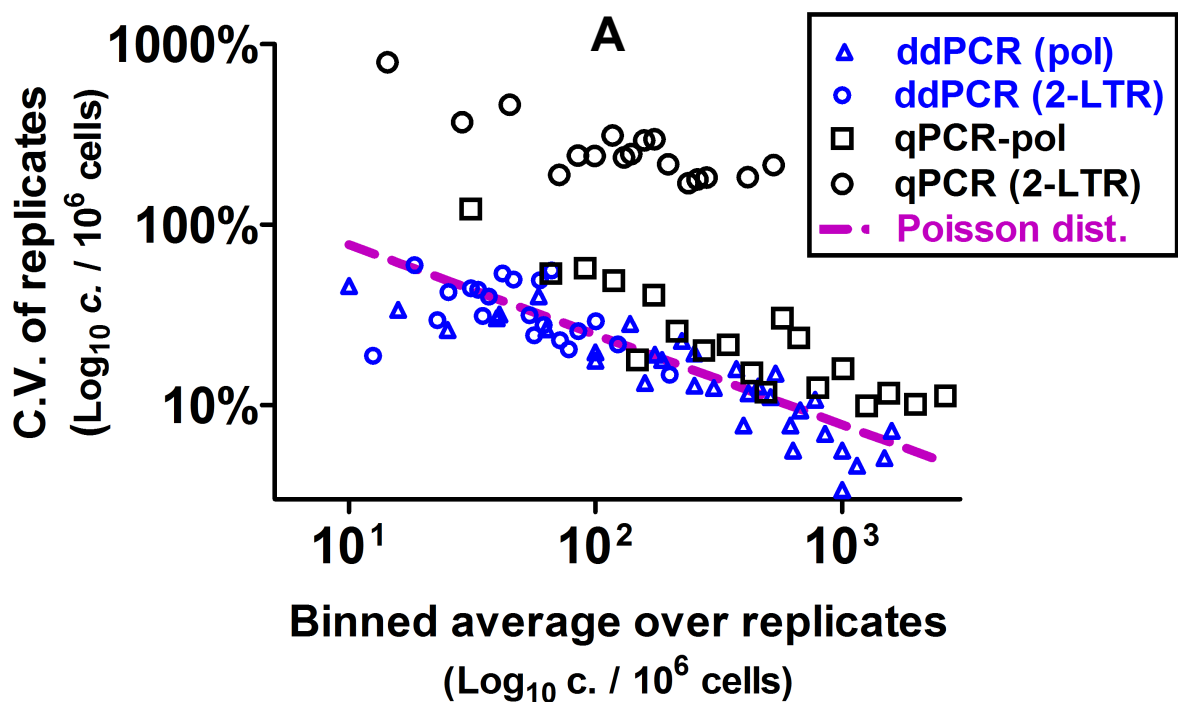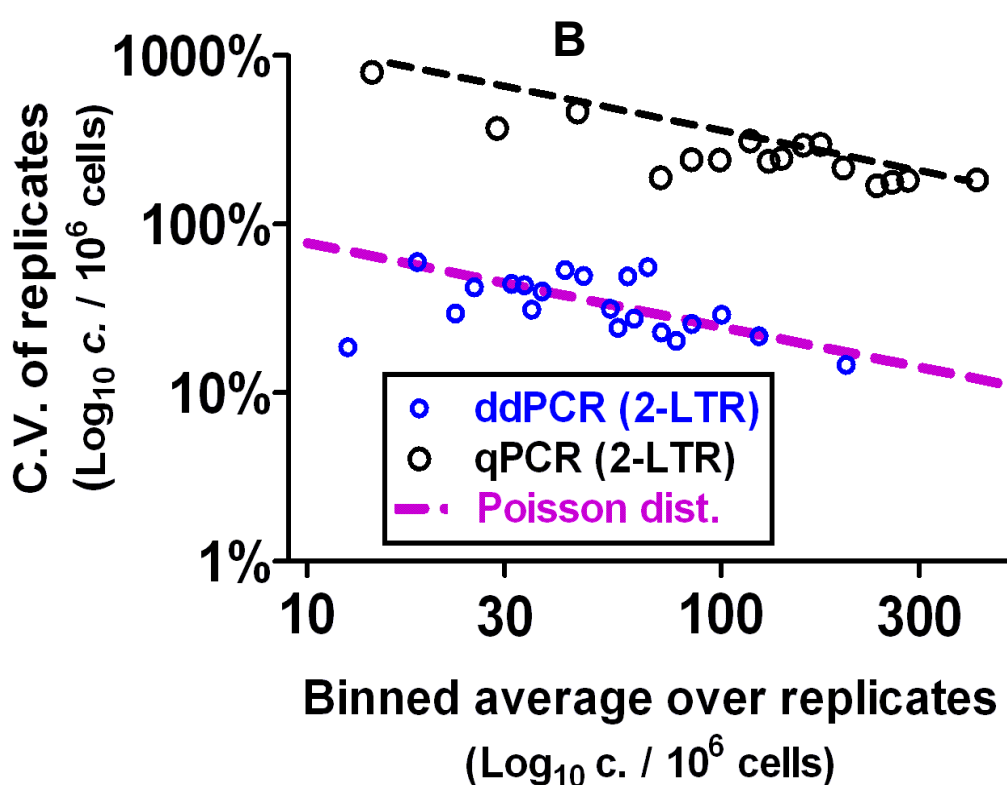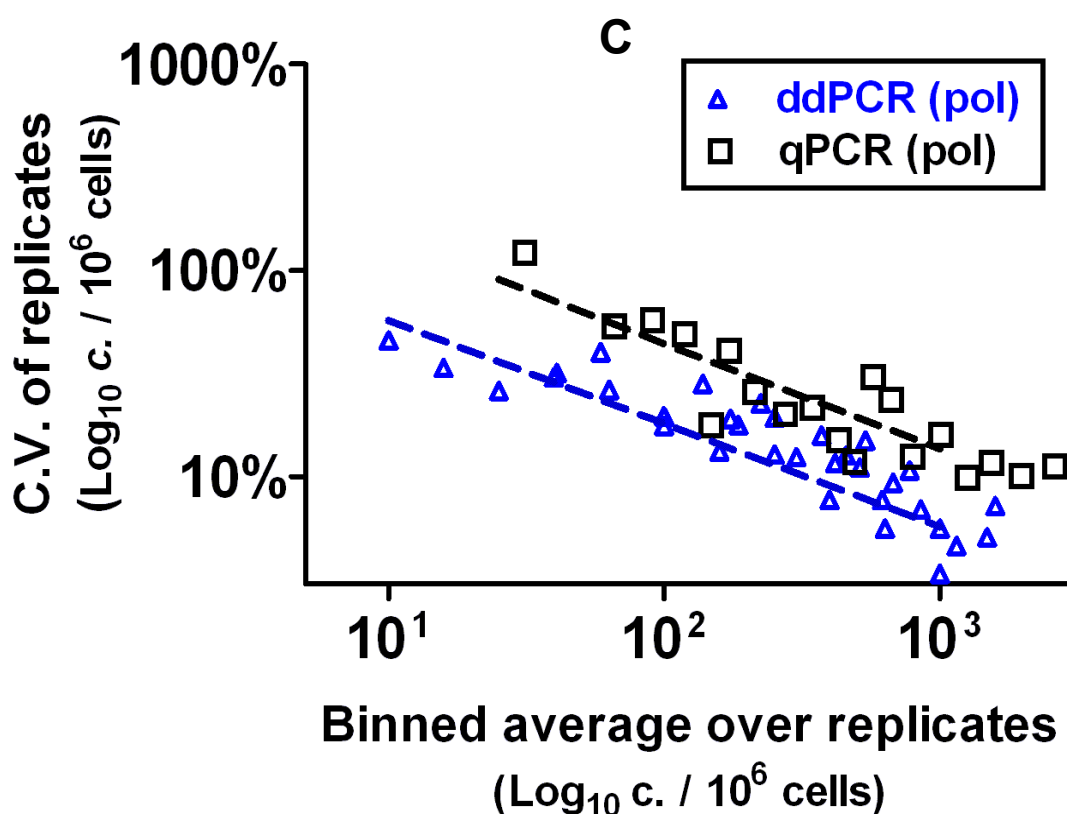

Supplement: Figure S4 — The ddPCR assay is more precise, particularly for 2-LTR circles. Samples isolated from the PBMC of infected patients were assayed by both methods. To compare the precision throughout the tested range, the C.V. was averaged over bins as in Fig. S2 (b). All 370 clinical samples analyzed by ddPCR in Figs. 3 and S2 (a) were included, despite the fact that qPCR data was only available for the 156 samples shown in Fig. 3. (a) For both methods and both targets, the C.V. increases with a slope statistically that is not significantly different from ½, the expected value for Poisson-distributed noise (dashed line). Trend lines computed independently for the four assays did not have distinguishable slopes. Therefore, in order to estimate the average relative precision, maximum likelihood fits shown assume the ½ exponent. The offset between the trend lines indicates the relative precision. (b) For the pol target, ddPCR is 4-fold more precise. (c) The precision improvement is much greater for the 2-LTR target, with an average 20-fold improvement over qPCR. (PDF) [file pone.0055943.s004.pdf]

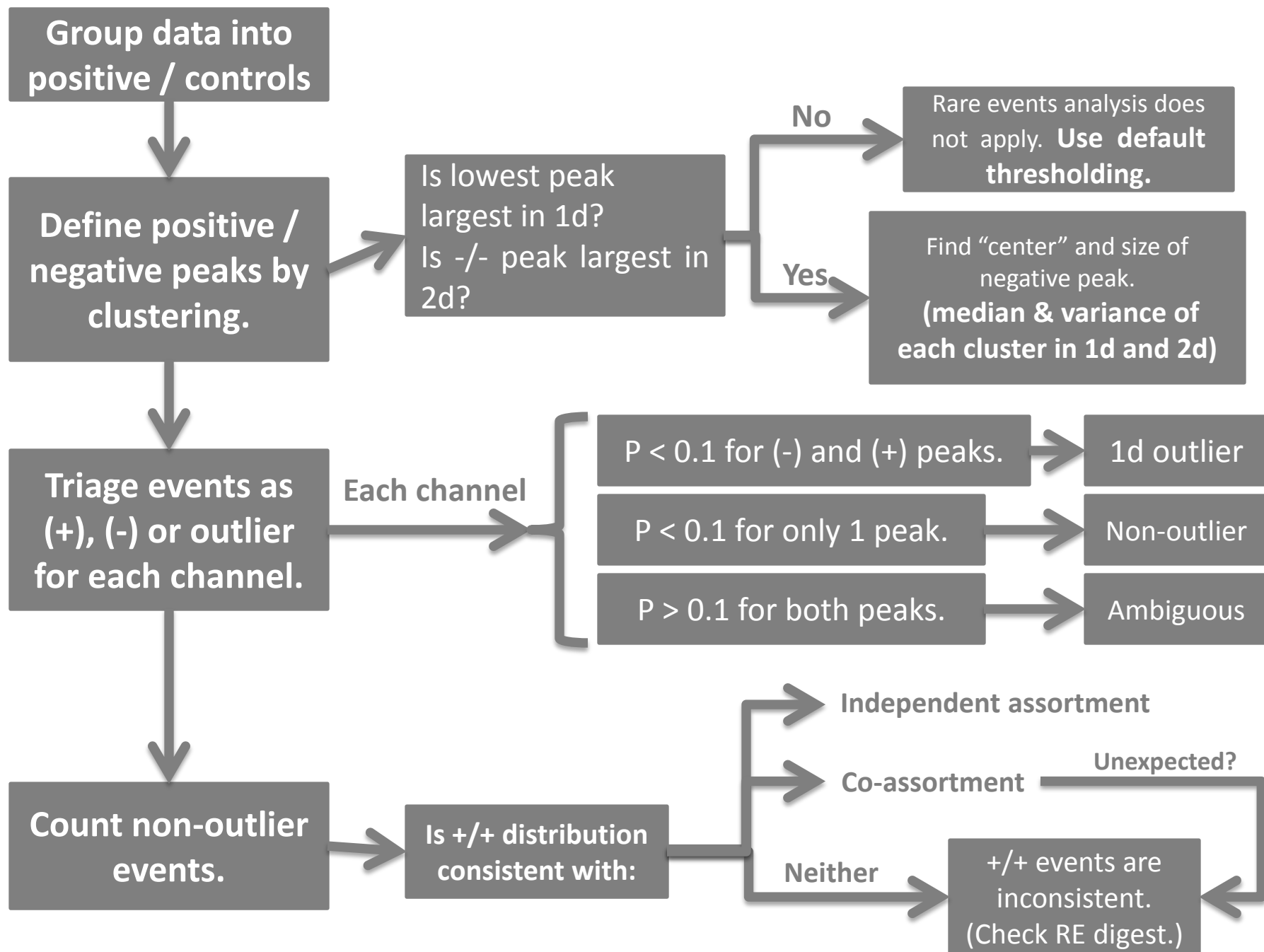

Supplement: Figure S5 — Triage classification of events. Raw fluorescence data were first filtered to eliminate events consistent with irregular droplet size (“rain” and “hail”). The remaining events were analyzed according to the algorithm shown. First, the largest droplet clusters were identified. In most cases, positive events were rare and nearly all events were associated with droplets that were negative in both fluorescence channels. Significant clusters were then approximated with a binormal distribution, and the probability of each droplet was determined for each of these distributions. Events that were highly unlikely within any of the binormal distributions were classified as ambiguous. Finally, independent assortment of the duplexed targets was used to eliminate events with an unlikely combination of fluorescence amplitudes. Restriction enzymes used in this study were always expected to cut between the duplexed amplicons, so the number of positive events in each channel was assumed independent. This was used to identify spurious double-positive events. (PDF) [file pone.0055943.s005.pdf]

**A**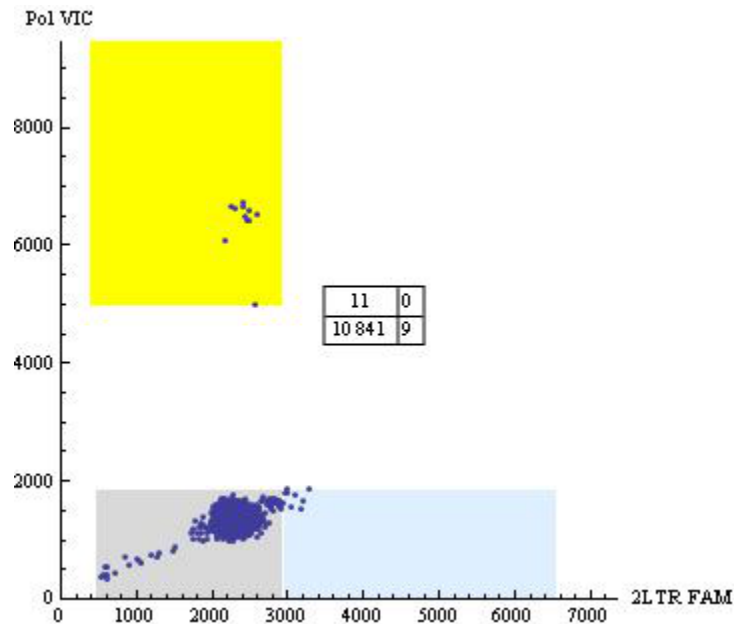**B**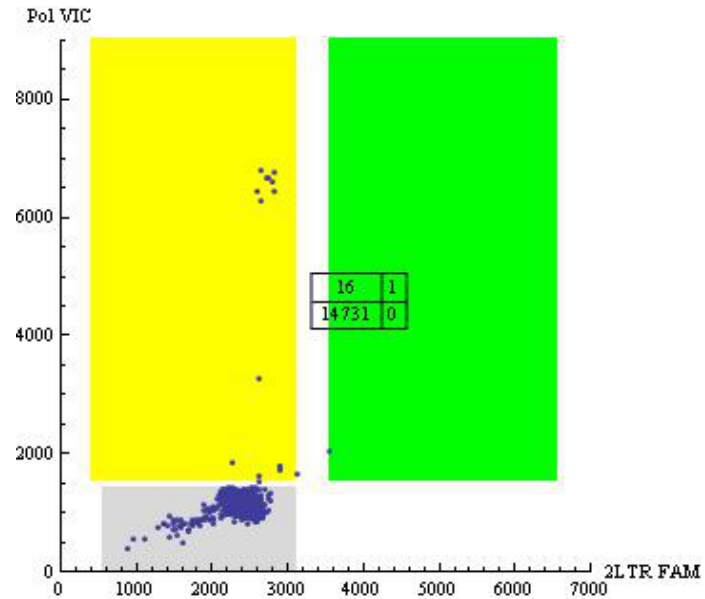

Supplement: Figure S6 — Sample dot-plots illustrate threshold ambiguities in ddPCR. Raw fluorescence values from a single well are shown. Default thresholds set by Bio-Rad QuantaSoft analysis software (version 1.1) are shown as colored rectangles, and the corresponding event counts in each quadrant are shown. (a) “Rain” and “hail” extend outward from the central peak of dual-negative events, but these are easily distinguished from true positive events by cluster analysis. (b) The pattern is similar to (a), but the sparseness of hail complicates its discrimination from true positive events. The one dual-positive event called by QuantaSoft can be eliminated by the assumption of independent assortment. (PDF) [file pone.0055943.s006.pdf]

**A**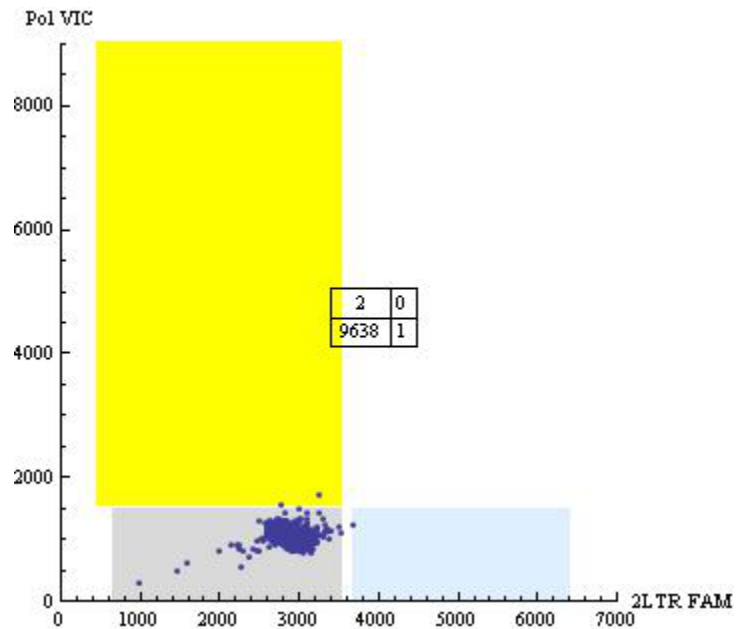**B**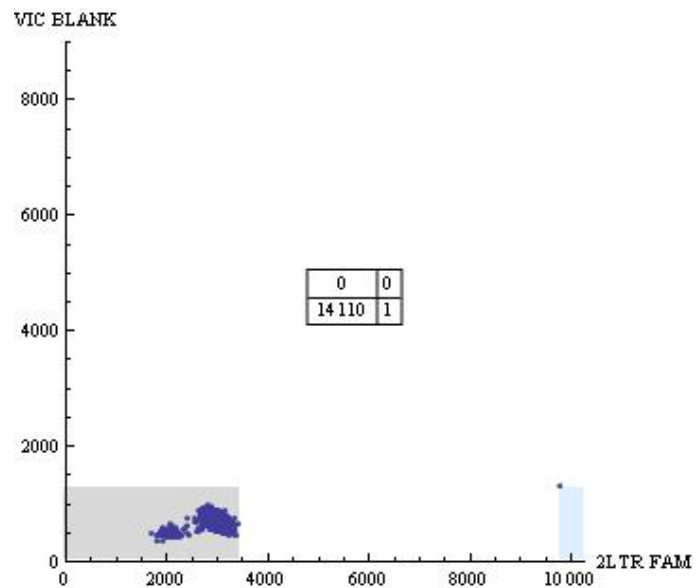

Supplement: Figure S7 — Sample false positives in ddPCR. Positive events called in no-template control wells may reflect erroneous calls by the default thresholding algorithm (a), but most false-positive events are well separated from true negative events and thus cannot be identified mathematically (b). (PDF) [file pone.0055943.s007.pdf]
