# Supplementary material for: Highly Precise Measurement of HIV DNA by Droplet Digital PCR
Source: PLoS One. 2013 Apr 3;8(4):e55943. doi: 10.1371/journal.pone.0055943 (PMC3616050; doi:10.1371/journal.pone.0055943)
Supplement: Table S1 — PCR Primers and Probes. (DOCX) [file pone.0055943.s008.docx]

| HIV pol forward (mf299) | GCA CTT TAA ATT TTC CCA TTA GTC CTA |
| --- | --- |
| HIV pol reverse (mf302) | CAA ATT TCT ACT AAT GCT TTT ATT TTT TC |
| HIV pol probe (mf348) | VIC-AAG CCA GGA ATG GAT GGC C-MGB |
| 2-LTR circle forward (MH535) | AAC TAG GGA ACC CAC TGC TTA AG |
| 2-LTR reverse (MH536) | TCC ACA GAT CAA GGA TAT CTT GTC |
| 2-LTR probe (MH603) | FAM-ACA CTA CTT GAA GCA CTC AAG GCA AGC TTT-MGB |
| RPP30 forward | GAT TTG GAC CTG CGA GCG |
| RPP30 reverse | GCG GCT GTC TCC ACA AGT |
| RPP30 probe | VIC-CTG ACC TGA AGG CTC T-MGB |
