# Supplementary material for: Highly Precise Measurement of HIV DNA by Droplet Digital PCR
Source: PLoS One. 2013 Apr 3;8(4):e55943. doi: 10.1371/journal.pone.0055943 (PMC3616050; doi:10.1371/journal.pone.0055943)
Supplement: Table S2 — Summary of Clinical Characteristics for Unblinded Patients. The 156 samples analyzed by both qPCR and ddPCR were drawn from ACTG5248, during the first 6 months of treatment. (DOCX) [file pone.0055943.s009.docx]

| **Number of patients** | 24 |
| --- | --- |
| **Time on ART (days)** | 0, 2, 7, (14), 28, 90, 180  (for each patient) |
| **Median viral load** | 131 |
| **Time points with [HIV RNA] < 50 c/ml** | 40% |
| **cART Regimen** | efavirenz (N=12) or raltegravir (N=12) +  tenofovir / emtricitabine |
